# Can deep transcranial magnetic stimulation (DTMS) be used to treat substance use disorders (SUD)? A systematic review

**DOI:** 10.1186/s12888-018-1704-0

**Published:** 2018-05-18

**Authors:** Karina Karolina Kedzior, Imke Gerkensmeier, Maria Schuchinsky

**Affiliations:** 1 Institute of Psychology and Transfer, University of Bremen, Bremen, Germany; 2 University of Limerick, Limerick, Ireland

**Keywords:** Substance use disorders (SUD), Deep transcranial magnetic stimulation (DTMS), H-coil, Alcohol, Nicotine, Cocaine

## Abstract

**Background:**

Deep transcranial magnetic stimulation (DTMS) is a non-invasive method of stimulating widespread cortical areas and, presumably, deeper neural networks. The current study assessed the effects of DTMS in the treatment of substance use disorders (SUD) using a systematic review.

**Methods:**

Electronic literature search (PsycInfo, Medline until April 2017) identified *k* = 9 studies (*k* = 4 randomized-controlled trials, RCTs, with inactive sham and *k* = 5 open-label studies). DTMS was most commonly applied using high frequency/intensity (10–20 Hz/100–120% of the resting motor threshold, MT) protocols for 10–20 daily sessions in cases with alcohol, nicotine or cocaine use disorders. The outcome measures were craving and dependence (according to standardized scales) or consumption (frequency, abstinence or results of biological assays) at the end of the daily treatment phases and at the last follow-up.

**Results:**

Acute and longer-term (6–12 months) reductions in alcohol craving were observed after 20 sessions (20 Hz, 120% MT) relative to baseline in *k* = 4 open-label studies with comorbid SUD and major depressive disorder (MDD). In *k* = 2 RCTs without MDD, alcohol consumption acutely decreased after 10–12 sessions (10–20 Hz, 100–120% MT) relative to baseline or to sham. Alcohol craving was reduced only after higher frequency/intensity DTMS (20 Hz, 120% MT) relative to sham in *k* = 1 RCT. Nicotine consumption was reduced and abstinence was increased after 13 sessions (10 Hz, 120% MT) and at the 6-month follow-up relative to sham in *k* = 1 RCT. Cocaine craving was reduced after 12 sessions (15 Hz, 100% MT) and at the 2-month follow-up relative to baseline in *k* = 1 open-label study while consumption was reduced after 12 sessions (10 Hz, 100% MT) relative to baseline but not to sham in *k* = 1 RCT.

**Conclusions:**

High-frequency DTMS may be effective at treating some SUD both acutely and in the longer-term. Large RCTs with inactive sham are required to determine the efficacy and the optimal stimulation parameters of DTMS for the treatment of SUD.

## Background

Deep transcranial magnetic stimulation (DTMS) with the H-coil system is a relatively novel non-invasive brain stimulation method [1]. Since 2013 DTMS is approved by the US Food and Drug Administration (FDA) for treatment-resistant unipolar major depression (MDD). The most commonly utilized DTMS protocol involves high-frequency (18–20 Hz) and high intensity (120% of the resting motor threshold, MT) stimulation delivered for 20 days. Such protocol has acute antidepressant [2, 3] as well as anxiolytic [4] properties in MDD, and tends to improve working memory and executive functioning in MDD and schizophrenia [5, 6].

The current focus in the field is to test other therapeutic applications of DTMS beyond MDD [7, 8]. One such application involves the use of DTMS for treatment of substance use disorders (SUD). Since SUD are difficult to treat with only modest responses to available therapies [9], they increase the burden of disease, in particular in comorbid conditions [10]. The evidence to date suggests that depending on the protocols and the coils, the non-invasive, repetitive brain stimulation methods appear to affect the neuroplasticity around the coil as well as throughout the brain [9, 11]. The functional reorganization could affect behaviors related to addiction, including reduction in craving and a better regulation of the compulsive desire to consume the substances [9, 11]. The unique structure of the H-coils is of particular interest because it allows to repetitively stimulate the entire cortex in contrast to other systems offering a more focal stimulation (for example, the figure of eight, F8-coil, used for repetitive transcranial magnetic stimulation, rTMS). Such a broad stimulation with H-coils could increase the electrical field in deeper, subcortical brain regions [12, 13]. Therefore, DTMS with H-coils is a promising candidate for treatment of various SUD that affect similar neural circuitry (deeper cortico-striatal pathways) [14] and share numerous molecular targets [15].

The aim of the current study was to systematically assess the effects of DTMS in the treatment of SUD.

## Methods

The current review was conducted according to the Preferred Reporting Items for Systematic Reviews and Meta-Analyses (PRISMA) guidelines [16].

### Systematic search strategy and study selection

An electronic search of PsycInfo, Medline, and Google Scholar (until April 2017) identified *k* = 133 studies (Table 1).

**Table 1:** Systematic search strategy for primary studies

*k* studies	Search terms	Databases (time frame)
133	TI (“deep transcranial magnetic stimulation” OR “deep repetitive transcranial magnetic stimulation” OR deep rTMS OR deepTMS OR deep TMS OR H-coil)	PsycInfo, Medline (OVID) (any date – 28.04.2017)

Search was performed in English with no language restrictions or any other limits

*k* number of studies, *rTMS* repetitive transcranial magnetic stimulation, *TI* title, *TMS* transcranial magnetic stimulation

Nine studies [17–25] met the following inclusion criteria:

1. DTMS applied in cases with any SUD using any type of H-coil,
2. any study designs including double-blind randomized controlled trials (RCTs) with inactive sham groups or open-label designs,
3. parallel designs in case of RCTs (to prevent any carry-over effects),
4. any number of cases/study (including single case studies),
5. SUD assessed at baseline and after DTMS using any method (standardized scales or biological assays).

**Fig. 1:**
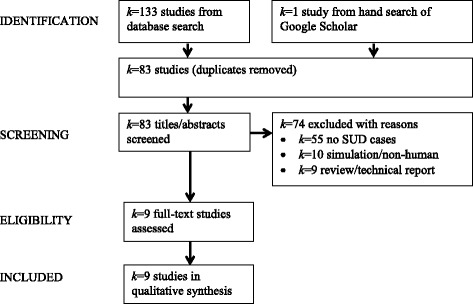
Study selection procedure (PRISMA flowchart). Note. Abbreviations: *k*, number of studies; SUD, substance use disorders

Studies were excluded if they did not assess SUD (*k* = 55/83), did not include human data (*k* = 10/83) or were reviews (*k* = 9/83; Fig. 1).

### Data coding and outcome measures

Data from *k* = 9 studies were coded by the authors independently and any inconsistencies were resolved by consensus. Study details (participant characteristics, study designs, SUD assessment methods, and stimulation parameters) are shown in Tables 2, 3 and 4.

**Table 2:** Participant characteristics in *k* = 9 studies

Study; country	*n* ^*a*^	Age (years) Mean ± *SD*	Male *n* (%)	Use duration (years) Mean ± *SD*	Abstinence period	SUD, Comorbid diagnosis	Diagnostic system	Dropout rate acute (daily) phase *n* (%)
Alcohol								
Rapinesi et al., 2013 [17]; Italy	3	53 ± 6	2 (67%)	28 ± 15	1 month	AUD, DD	DSM-IV-TR	0 (0%)
Rapinesi et al., 2014 [19]; Italy	1	56	1 (100%)	1	none	AUD, MDD suicidal ideation	–	0 (0%)
Ceccanti et al., 2015 [20]; Italy	9 DTMS 9 sham	43 ± 11 47 ± 11	9 (100%) 9 (100%)	26 ± 9 25 ± 11	11 days	AUD	DSM-IV	4/9 (44%)^b^ 3/9 (33%)
Girardi et al., 2015 [21]; Italy	10	53 ± 8	5 (50%)	10 ± 5	1 month	AUD, DD	DSM-IV-TR	0 (0%)
Rapinesi et al., 2015 [22]; Italy	11	54 ± 8	6 (54%)	19 ± 8	1 month	AUD, MDD	DSM-IV-TR	0 (0%)
Addolorato et al., 2017 [25]; Italy	5 DTMS 6 sham	–	4 (80%) 5 (83%)	–	none	AUD	DSM-V	0 (0%)
Nicotine								
Dinur-Klein et al., 2014 [18]; Israel	16 (10 Hz + cue) 16 (10 Hz –cue) 7 (1 Hz + cue) 7 (1 Hz –cue) 15 (sham +cue) 16 (sham –cue)	50 ± 12 50 ± 9 48 ± 11 50 ± 12 52 ± 11 50 ± 8	11 (69%) 12 (75%) 4 (57%) 3 (43%) 10 (67%) 8 (50%)	42 ± 16 43 ± 21 40 ± 24 46 ± 26 37 ± 14 42 ± 14	≥1 h	none	–	38/115 (33%)^c^
Cocaine								
Bolloni et al., 2016 [24]; Italy	6 DTMS 4 sham	35 34	5 (83%) 3 (75%)	13 12	–	CUD	DSM-V	4/10 (40%) 4/8 (50%)
Rapinesi et al., 2016 [23]; Italy	7	49 ± 9	7 (100%)	16 ± 11	–	CUD	DSM-IV-TR	0 (0%)

^a^Sample size in groups with SUDs who received DTMS or sham

^b^Dropout rate during 1–2 months of the study (during the acute and follow-up phases)

^c^Dropout rate from all groups (acute phase)

*AUD* alcohol use disorder, *CUD* cocaine use disorder, *DD* dysthymic disorder, *DTMS* deep transcranial magnetic stimulation, *k* number of studies, *MDD* major depressive disorder, *n* sample size, *SD* standard deviation, *SUD* substance use disorder

**Table 3:** Study design and SUD assessment in *k* = 9 studies

Study	SUD assessment	Acute (after daily DTMS) assessment	Last follow-up assessment^a^	Treatment strategy^b^	Study design	Study groups
Alcohol						
Rapinesi et al., 2013 [17]	OCDS	Session 20	6 months	add-on	open-label (case study)	1 group: DTMS
Rapinesi et al., 2014 [19]	OCDS	Session 20	12 months	add-on	open-label (case study)	1 group: DTMS
Ceccanti et al., 2015 [20]	VAS, TLFB, blood prolactin	Session 10	2–3 months	monotherapy	RCT	2 groups (both with cue condition^c^): DTMS vs. sham
Girardi et al., 2015 [21]	OCDS	Session 20	6 months	add-on	open-label	2 groups: DTMS vs. STD (no DTMS)
Rapinesi et al., 2015 [22]	OCDS	Session 20	6 months	add-on	open-label	2 groups: DTMS (MDD + AUD) vs. DTMS (MDD)
Addolorato et al., 2017 [25]	OCDS, ADS, TLFB, SPECT	Session 12	–	monotherapy	RCT	3 groups: DTMS vs. sham vs. healthy (no DTMS)
Nicotine						
Dinur-Klein et al., 2014 [18]	FTND, sTCQ, urine analysis	Session 13	6 months	monotherapy	RCT	6 groups (each with or without cue condition^c^): DTMS (10 Hz) vs. DTMS (1 Hz) vs. sham
Cocaine						
Bolloni et al., 2016 [24]	Hair analysis	Session 12	6 months	monotherapy	RCT	2 groups: DTMS vs. sham
Rapinesi et al., 2016 [23]	VAS	Session 12	2 months	add-on	open-label	1 group: DTMS

^a^There was no maintenance DTMS treatment during the follow-up phases

^b^Add-on means that DTMS was administered in addition to concurrent pharmacotherapy

^c^The cue condition consisted of a substance-related stimulus (e.g. raising a glass filled with a favourite alcoholic drink) prior to DTMS

*ADS* Alcohol Dependence Scale [41], *DTMS* deep transcranial magnetic stimulation, *FTND* Fagerström Test for Nicotine Dependence [42], *HDRS* Hamilton Depression Rating Scale [43], *k* number of studies, *OCDS* Obsessive Compulsive Drinking Scale [44], *RCT* double-blind randomised controlled trial with inactive sham control group, *SDT* standard drug treatment, *SPECT* single photon emission computed tomography, *sTCQ* the short Tobacco Craving Questionnaire [45], *SUD* substance use disorder, *TLFB* Timeline Followback Interview (assessment of consumption) [46], *VAS* visual analogue scale for craving [47]

**Table 4:** Stimulation parameters during acute (daily DTMS) phases in *k* = 9 studies

Study	Coil type^a^	Stimulation location^b^	Frequency (Hz)	Intensity (% MT)	Total stimuli	Stimuli/session	Trains/session	Inter-train interval (s)	No. of sessions
Alcohol									
Rapinesi et al., 2013 [17]	H1	5.5 cm	20	120	–	–	55	20	20
Rapinesi et al., 2014 [19]	H1	5.5 cm	18	120	–	–	55	20	20
Ceccanti et al., 2015 [20]^c^	H1	5 cm	20	120	15,000	1500	30	30	10
Girardi et al., 2015 [21]	H1	5.5 cm	20	120	–	–	55	20	20
Rapinesi et al., 2015 [22]	H1	5.5 cm	18	120	39,600	1980	55	20	20
Addolorato et al., 2017 [25]^c^	H	5.5 cm	10	100	–	–	20	15	12
Nicotine									
Dinur-Klein et al., 2014 [18]^c^	HADD	6 cm	10	120	12,870	990	33	20	13
	HADD	6 cm	1	120	7800	600	33	20	13
Cocaine									
Bolloni et al., 2016 [24]^c^	H1	–	10	100	–	–	20	15	12
Rapinesi et al., 2016 [23]	H1	5.5 cm	15	100	–	–	20	20	12

^a^Prefrontal cortex was stimulated in all studies

^b^Distance from the motor ‘hot-spot’

^c^Active stimulation parameters

*DTMS* deep transcranial magnetic stimulation, *k* number of studies, *MT* resting motor threshold

The primary outcome measures were craving and dependence (assessed with standardized scales listed in Table 3) or consumption (use frequency, abstinence rate or results of urine, blood or hair analysis; Table 3) at the end of the acute (daily) treatment with DTMS and at the last follow-up. Since some studies included samples with comorbid MDD, the secondary outcome measure was depression severity according to the Hamilton Depression Rating Scale (HDRS).

### Risk of bias assessment (RCTs)

The risk of bias in RCTs was assessed using the criteria proposed by Cochrane [26]. The outcomes of this assessment for each individual study are shown in Table 5.

**Table 5:** Cochrane risk of bias assessment in *k* = 4 RCTs

Study	Random sequence generation (selection bias)	Allocation concealment selection bias)	Blinding of participants and personnel (performance bias)	Blinding of outcome assessors (detection bias)	Incomplete outcome data (attrition bias)	Selective reporting (reporting bias)	Other bias
Dinur-Klein et al., 2014 [18]	LOW	LOW	LOW	LOW	LOW	LOW	HIGH
Ceccanti et al., 2015 [20]	LOW	LOW	LOW	LOW	UNCLEAR	LOW	HIGH
Bolloni et al., 2016 [24]	UNCLEAR	UNCLEAR	LOW	LOW	HIGH	LOW	LOW
Addolorato et al., 2017 [25]	LOW	LOW	LOW	LOW	LOW	LOW	LOW

## Results

### Study details (Tables 2 and 3)

Our review comprises *k* = 9 studies including *k* = 5 studies with open-label designs and *k* = 4 double-blind RCTs with sham control groups. Of all 148 participants, *n* = 84 received active high-frequency (10–20 Hz) DTMS, *n* = 50 sham treatment, and *n* = 14 low-frequency (1 Hz) DTMS. Most participants in all studies were middle-aged and male. Substance-related diagnoses included alcohol use disorders (AUD) in *k* = 6 studies (*n* = 39 DTMS, *n* = 15 sham), nicotine dependence (*k* = 1; *n* = 32 high-frequency DTMS, *n* = 14 low-frequency DTMS, *n* = 31 sham), and cocaine use disorders (CUD; *k* = 2, *n* = 13 DTMS, *n* = 4 sham). The participants in all studies were seeking treatment for their SUD and were recruited from the general population or from clinics specializing in SUD. Most were heavy, long-term users of alcohol, nicotine or cocaine (average use of 10–46 years) who either did not successfully respond to or relapsed after other treatments. DTMS was administered either as an add-on therapy to antidepressants and/or to mood stabilizers in *k* = 5 open-label studies or as a monotherapy in *k* = 4 RCTs.

The last follow-up assessments were conducted at 2–12 months after the last acute DTMS sessions. Once the course of daily stimulation ended, there was no maintenance DTMS treatment in any of the studies. Substance-related cues were presented before DTMS in two studies with AUD or nicotine use disorders, respectively [18, 20].

### Stimulation parameters (Table 4)

The majority of studies utilized H1 coil (*k* = 7) and a high frequency/intensity (10–20 Hz/100–120% of the resting motor threshold, MT) stimulation protocol applied for 10–20 acute (daily or almost daily) sessions. One study [18] also included a low-frequency (1 Hz) stimulation protocol with 120% MT and 13 sessions.

### Risk of bias in the RCTs (Table 5)

The quality of the *k* = 4 RCTs was acceptable in terms of randomization, blinding, and reporting of results. However, the outcome assessment was problematic (underpowered) in three RCTs due to small sample sizes and high attrition. Furthermore, two of the four RCTs were industry-sponsored.

### DTMS and SUD (Table 6)

The results synthesized from all *k* = 9 studies suggest that overall the high-frequency DTMS was effective at treating various symptoms of different SUD, both acutely and in the longer term (Table 6).

**Table 6:** Acute and longer-terms effects of DTMS on SUD: synthesis of results from *k* = 9 studies

Study; *n*^a^	Substance/ SUD^b^/ Scale	Study design/ Treatment^c^	Coil/ Frequency /Intensity (%MT)	Primary outcomes		Secondary outcomes (acute & at follow-up)^e^
				Acute (after daily DTMS)	Last follow-up (months since DTMS)^d^	
Rapinesi et al., 2013 [17]; *n* = 3	Alcohol/ AUD/ OCDS	Open-label/ Add-on	H1/ 20 Hz/ 120%	Post-DTMS (20 sessions) vs. baseline:	Post-DTMS (6 months) vs. baseline:	↓ HDRS
				↓ craving	↓ craving	
Rapinesi et al., 2014 [19]; *n* = 1	Alcohol/ AUD/ OCDS	Open-label/ Add-on	H1/ 18 Hz/ 120%	Post-DTMS (20 sessions) vs. baseline:	Post-DTMS (12 months) vs. baseline:	↓ HDRS
				↓ craving	↓ craving; ↑ abstinence	
Ceccanti et al., 2015 [20]^f^; *n* = 9 (*n* = 9 sham)	Alcohol/ AUD/ VAS	RCT/ Monotherapy	H1 (+cue)/ 20 Hz/ 120% (vs. sham)	Post-DTMS vs. sham (10 sessions):	Post-DTMS vs. sham (2–3 months):	–
				↓ craving, daily use/maximum use, blood cortisol (stress hormone), blood prolactin (marker of dopamine activity)	(trend) ↓ craving, daily use/maximum use	
				Post-DTMS (10 sessions) vs. baseline:	Post-DTMS (2–3 months) vs. baseline:	
				↓ craving, daily use/maximum use, blood cortisol and prolactin	(trend) ↓ craving, maximum use; ↓ daily use	
Girardi et al., 2015 [21]; *n* = 10	Alcohol/ AUD/ OCDS	Open-label/ Add-on	H1/ 20 Hz/ 120%	Post-DTMS (20 sessions) vs. baseline:	Post-DTMS (6 months) vs. baseline:	↓ HDRS
				↓ craving	↓ craving	
Rapinesi et al., 2015 [22]; *n* = 11	Alcohol/ AUD/ OCDS	Open-label/ Add-on	H1/ 18 Hz/ 120%	Post-DTMS (20 sessions) vs. baseline:	Post-DTMS (6 months) vs. baseline:	↓ HDRS
				↓ craving	↓ craving	
Addolorato et al., 2017 [25]; *n* = 5 (*n* = 6 sham)	Alcohol/ AUD/ OCDS	RCT/ Monotherapy	H/ 10 Hz/ 100% (vs. sham)	Post-DTMS (12 sessions) vs. baseline:	–	–
				↔ craving; ↓ daily use/total use, striatal dopamine transporter (SPECT); ↑ abstinence		
				Post-sham (12 sessions) vs. baseline:		
				↔ all effects (no change from baseline)		
Dinur-Klein et al., 2014 [18]^f^; 10 Hz + cue: *n* = 16 (*n* = 15 sham); 10 Hz–cue: *n* = 16 (*n* = 16 sham); 1 Hz + cue: *n* = 7; 1 Hz–cue: *n* = 7	Nicotine/ no SUD/ sTCQ; FTND	RCT/ Monotherapy	HADD (+ or –cue)/ 10 Hz/ 120%/ vs. 1 Hz/ 120% (vs. sham)	Post-DTMS (10 Hz) vs. sham (13 sessions):	Post-DTMS (10 Hz) vs. sham (6 months):	–
				(trend) ↑ efficacy +cue vs. –cue; ↔ craving; ↓ cigarettes/day, dependence, consumption (urine cotinine); ↑ abstinence (25–44% vs. 0–13% sham), response (50% ↓ in consumption); 1 Hz DTMS discontinued due to poor efficacy	↓ cigarettes/day; ↑ abstinence (23–33% vs. 0–9% sham)	
Bolloni et al., 2016 [24]; *n* = 6 (*n* = 4 sham)	Cocaine/ CUD/ no scale	RCT/ Monotherapy	H1/ 10 Hz/ 100% (vs. sham)	Post-DTMS vs. sham (12 sessions):	Post-DTMS vs. sham (6 months):	–
				↔ consumption (hair cocaine)	↔ consumption (hair cocaine)	
				Post-DTMS (12 sessions) vs. baseline:	Post-DTMS (6 months) vs. baseline:	
				↓ consumption (hair cocaine)	↓ consumption (hair cocaine)	
Rapinesi et al., 2016 [23]; *n* = 7	Cocaine/ CUD/ VAS	Open-label/ Add-on	H1/ 15 Hz/ 100%	Post-DTMS (12 sessions) vs. baseline:	Post-DTMS (2 months) vs. baseline:	–
				↓ craving	↓ craving	

*Abbreviations*: *AUD* alcohol use disorder, *CUD* cocaine use disorder, *DTMS* deep transcranial magnetic stimulation, *FTND* Fagerstrom Test for Nicotine Dependence, *HDRS* Hamilton Depression Rating Scale, *k* number of studies, *MT* resting motor threshold, *n* sample size at baseline in DTMS groups, *OCDS* Obsessive Compulsive Drinking Scale, *RCT* double-blind randomized controlled trial with inactive sham control group, *SPECT* single photon emission computed tomography, *sTCQ* the short Tobacco Craving Questionnaire, *SUD* substance use disorder (dependence and/or abuse), *VAS* visual analogue scale for craving

^a^Cases with SUD who received DTMS; ^b^SUD according to DSM-IV, −TR or –V; ^c^Add-on means DTMS with concurrent pharmacotherapy; ^d^No maintenance DTMS during the follow-up phases; ^e^Studies with comorbid SUD and MDD or dysthymic disorder (DSM-IV-TR); ^f^Cue conditions were substance-related stimuli presented before DTMS

### Alcohol outcomes

DTMS with H1-coil consistently alleviated AUD symptoms, particularly in *k* = 4 open-label studies with comorbid AUD and MDD. Specifically, alcohol craving and/or urge was acutely reduced after DTMS (20 sessions/18–20 Hz/120% MT) relative to baseline and at follow-up (6–12 months) relative to baseline. In addition, depression severity was also alleviated, both acutely and at follow-up (6–12 months) relative to baseline in all *k* = 4 studies.

The acute effects of DTMS were also reported in *k* = 2 RCTs with inactive sham that included AUD cases without MDD [20, 25]. Daily use was reduced in both RCTs after DTMS relative to baseline or to sham. However, alcohol craving was acutely reduced in only one of the RCTs [20] with a higher frequency/intensity protocol (10 sessions/20 Hz/120% MT) relative to baseline or to sham. The effects of active DTMS relative to sham were not computed and no change in craving was acutely observed after DTMS with lower frequency/intensity (12 sessions/10 Hz/100% MT) relative to baseline in the other RCT [25]. Although the longer-term effects of DTMS on AUD could not be reliably established due to a high drop-out rate in one RCT [20], craving and consumption tended to remain lower in the active DTMS group at follow-up (2–3 months) relative to baseline or to sham.

In addition to the subjective assessment of SUD symptoms (with scales or self-reported frequency of use), both RCTs also reported acutely reduced dopamine activity using biological measures. Specifically, blood cortisol and prolactin levels (markers of dopamine activity) were reduced after DTMS relative to baseline or to sham [20] and striatal dopamine transporter (DAT) availability (measured with the single photon emission computed tomography, SPECT) was reduced after DTMS relative to baseline [25].

### Nicotine outcomes

Only one study (RCT with inactive sham) [18] assessed the effects of DTMS with HADD-coil on nicotine use. Although nicotine craving did not differ acutely after DTMS relative to sham, acute nicotine consumption and dependence were reduced and abstinence increased in the high-frequency DTMS groups (13 sessions/10 Hz/120% MT), in particular when DTMS was preceded by a smoking-related cue, relative to sham [18]. The reduction in nicotine consumption and continuous abstinence were also observed in the high-frequency DTMS groups at follow-up (6 months) relative to sham [18]. In contrast, low-frequency DTMS (1 Hz) had poor efficacy and was discontinued [18].

### Cocaine outcomes

The effects of DTMS with H1-coil on CUD were assessed in *k* = 2 studies (one RCT [24] and one open-label study [23]). Cocaine consumption (detected in hair) and craving were acutely reduced after DTMS (12 sessions/10–15 Hz/100% MT) relative to baseline and at follow-up (2–6 months) relative to baseline. However, there was no difference in cocaine consumption between the active DTMS relative to sham in the RCT [24].

### Safety

All patients completed the treatment with DTMS in six out of *k* = 9 studies included in this review. However, high drop-out rates were reported in *k* = 3 studies (Table 2). The authors of these studies claimed that DTMS is safe because no severe side-effects occurred. Mild adverse reactions to DTMS (headaches, nausea, discomfort) were reported in nine participants in *k* = 2 studies [18, 24]. Others dropped out due to relapse in SUD or other factors unrelated to treatment, such as inconvenience and other personal reasons.

## Discussion

Our review summarizes the preliminary evidence suggesting that high-frequency DTMS might be a promising treatment for cases with SUD who failed to respond to other available treatments. According to data from nine studies, craving, dependence, and consumption of alcohol, nicotine, and cocaine were reduced after daily treatment with DTMS and some of these effects lasted for up to 12 months without maintenance treatment. Although highly interesting, the evidence to date should be interpreted with caution because it is based on studies with open-label designs or RCTs with small sample sizes. Furthermore, the interpretation of results in these studies is difficult due to heterogeneous stimulation protocols, different SUD and SUD outcomes, and high drop-out rates especially after cessation of daily treatment.

While the most optimal stimulation parameters for the treatment of SUD remain unknown [14, 27], the current review shows that various SUD symptoms were alleviated following the high-frequency stimulation protocols. In particular, protocols with higher frequencies/intensities (18–20 Hz, 120% MT) reduced alcohol craving and depression severity in comorbid AUD and MDD, both acutely (after 20 sessions) and at follow-up (6–12 months). Since such protocols produce consistent acute antidepressant effects in MDD with AUD [10] but also in MDD alone [2, 3], the acute reduction in AUD symptoms might have been secondary to the alleviation of MDD symptoms. However, the effects of DTMS cannot be secondary alone because two RCTs without comorbid MDD [20, 25] also reported acute reductions in AUD symptoms after high-frequency DTMS. Thus, high-frequency protocols may acutely reduce the severity of both conditions (MDD and AUD). Protocols with lower frequencies/intensities (10–15 Hz/100% MT) and less daily sessions (10–13) also acutely reduced some aspects of nicotine use and CUD symptoms although the longer-term durability of these effects remains unclear due to the low volume of data. In contrast to high-frequency, low-frequency DTMS (1 Hz) was ineffective at reducing nicotine use [18]. Similar to evidence from rTMS studies [9, 11, 28], the efficacy of DTMS in the treatment of SUD is likely to depend on the length of stimulation in combination with frequency, intensity as well as other stimulation parameters. For example, DTMS outcomes in SUD may depend on the number of stimuli that have been shown to influence the antidepressant outcomes of rTMS with F8-coil [29, 30]. Furthermore, craving for some substances may be reduced based on a complex mechanism requiring both excitation with the high-frequency stimulation (10 Hz) and inhibition with the low-frequency stimulation (1 Hz) according to preliminary evidence from an rTMS study with F8-coil in methamphetamine users [28]. A higher volume of primary data is required to investigate the most optimal parameters of DTMS required for the acute treatment of SUD using multivariate statistical methods. It is also necessary to test the longer-term efficacy of DTMS to improve treatment compliance and reduce relapse after daily treatment.

Although the general idea of applying non-invasive brain stimulation to treat SUD symptoms is not new [9, 11, 27, 31, 32], the mechanism of action of these methods, including DTMS, is still unclear. Past reviews have shown that the high-frequency rTMS with F8-coil is able to transiently reduce craving for various substances [9, 14, 32–35], possibly due to changes in the dopamine and glutamate activity in the cortico-striato-limbic systems implicated in SUD [9, 14, 15]. These changes may include increased dopaminergic release and/or improved dopaminergic binding in the striatum, although multiple networks and neurotransmitter systems are likely to be involved depending on the type of SUD [36]. In general, although various SUD affect common neural pathways, share common molecular targets, and have similar reinforcing effects, they may access the reward system via different mechanisms [15]. Advancing efficient translational research is required to find new pharmacological treatments for SUD [15]. The non-invasive brain stimulation could be a viable alternative to pharmacotherapy in the short-term and/or could offer a longer-term relief from SUD although it may require some individualization of protocols and/or stimulation sites rather than the ‘one-size-fits-all’ approach [11]. In fact, the unique shape of the H-coil that delivers a broad cortical and presumably deeper, subcortical stimulation [12, 37] may be particularly useful in the longer-term treatment of SUD [38]. Indeed, the current review shows that the effects of DTMS may last for up to 12 months after daily stimulation phases despite the absence of maintenance treatment. It has been suggested that repetitive stimulation is required for persistent and enduring plastic neuroadaptations [11, 31]. In fact, the most consistent and longer-lasting plastic changes in SUD may result from a combination of *repetitive*, *broad,* and (presumably) *deep* stimulation achieved using H-coils with high frequencies (18–20 Hz) and delivered for at least 20 sessions. Such broad and deep stimulation could affect both the multiple nodes of the executive control network and the limbic network implicated in craving [27]. The enhanced cognitive control could also contribute to efficacy of DTMS in comorbid conditions, such as MDD and SUD [36]. If DTMS improves cognition first [5], the recovery of executive functioning could allow patients to subsequently regain cognitive control over their substance craving, as well as their negative mood states [22]. Future research is required to investigate the neurobiological mechanisms and the interaction between the cognitive outcomes and the alleviation of SUD symptoms following DTMS. Furthermore, head-to-head studies directly comparing the effects of various coils (for example, F8-coil and H1-coil) in the treatment of SUD are required to investigate which approach is more effective.

While there is interest in finding new treatments for SUD, the quality of evidence from DTMS studies is only preliminary so far, similar to the evidence from rTMS studies [9, 11]. The current review suggests that apart from assessing various stimulation protocols and the durability of the acute effects, other issues should also be considered in the future DTMS research in SUD. First, large, double-blind RCTs with inactive sham are required to determine the efficacy of DTMS in the treatment of various SUD. The studies reviewed here used open-label designs (*k* = 5) while the four RCTs with sham groups had only moderate quality at best. All studies reported a relatively low volume of data (only up to 11 cases received active DTMS treatment in eight out of the nine studies) and/or high drop-out rates (33–50% in three out of the nine studies). Therefore, the majority of studies were underpowered and placebo and expectancy effects were not controlled for. A preliminary meta-analysis of the acute effects in studies included in the current review showed a large pooled reduction in craving and dependence after active DTMS relative to baseline (Hedges’ *g* = 2.65, 95% confidence interval: 1.28–4.02, *k* = 5 studies [39]). However, this pooled effect was not controlled for sham and there was a trend towards higher effects in open-label studies relative to the RCTs [39]. The effects of DTMS on SUD cannot be explained by placebo/expectancy effects alone for a number of reasons. Trends in the data suggest that DTMS was more effective than sham for some symptoms of alcohol and nicotine use disorders according to three RCTs reviewed here. Furthermore, the subjective reports regarding the reductions in SUD symptom severity after DTMS were confirmed with biological changes, including reductions in either dopamine activity or concentrations of nicotine and cocaine metabolites. Finally, despite the absence of maintenance treatment, eight studies reported that some acute effects of DTMS on SUD tended to last for 2–12 months after the last daily DTMS session. Second, future DTMS studies need to carefully choose the outcome measures. Most studies report the effects of non-invasive brain stimulation on craving rather than the actual use [32]. The subjective self-reports of substance use should also be verified with biological assays. Although substance use tends to be reliably and validly reported in anonymous research contexts [40], it may be underreported in the clinical practice. Third, the clinical characteristics of patients could affect the outcomes of DTMS studies in SUD. The efficacy of DTMS may depend on SUD severity, concurrent pharmacotherapy, and polysubstance use. Our review suggests that DTMS may be most effective in combination with pharmacotherapy for comorbid MDD and AUD or for cases with higher severity of SUD. In contrast, DTMS as a monotherapy or for cases with lower severity of SUD may produce less satisfactory outcomes, possibly leading to higher dropout rates. Polysubstance use also needs to be carefully controlled for because H-coils targeting different neural regions may be required for best efficacy depending on the substance and/or the type of SUD. Finally, although no severe reactions to DTMS were reported, a systematic assessment of DTMS safety in SUD is necessary in future research.

There are a number of other limitations in the current review. First, eight out of nine studies were conducted in Italy. The clinical outcomes of DTMS on SUD symptoms need to be tested in other institutions/countries. Second, the current review considered only published literature from two academic databases. Although it is unlikely that other published studies on the topic exist, currently ongoing studies may provide higher-quality evidence regarding the use of DTMS in the treatment of SUD. Third, the current review is descriptive only and does not report any effect sizes. We conducted a preliminary meta-analysis based on craving and dependence outcomes using data from *k* = 5 studies [39]. The effect sizes were based on highly heterogeneous data, including different study designs (open-label vs. RCT), SUD types, outcome measures, and stimulation parameters. Therefore, a larger volume of data is required for a more reliable meta-analysis of the effects of DTMS on SUD.

## Conclusions

In conclusion, the current review provides preliminary evidence that high-frequency DTMS may be effective at treating some SUD symptoms both acutely and in the longer-term (for 2–12 months after daily treatment). Large RCTs with inactive sham are required to determine the efficacy and the optimal stimulation parameters of DTMS for the treatment of different SUD.

## Abbreviations

AUD: Alcohol use disorder
CUD: Cocaine use disorder
DTMS: Deep transcranial magnetic stimulation
FTND: Fagerstrom test for nicotine dependence
HDRS: Hamilton Depression Rating Scale
*k*: Number of studies
MT: Resting motor threshold
*n*: Sample size at baseline in DTMS groups
OCDS: Obsessive compulsive drinking scale
PRISMA: Preferred reporting items for systematic reviews and meta-analyses
RCT: Double-blind randomized controlled trial with inactive sham control group
rTMS: Repetitive transcranial magnetic stimulation
SPECT: Single photon emission computed tomography
sTCQ: The short tobacco craving questionnaire
SUD: Substance use disorder (dependence and/or abuse)
VAS: Visual analogue scale for craving
